# Cluster Cooperation in Wireless-Powered Sensor Networks: Modeling and Performance Analysis

**DOI:** 10.3390/s17102215

**Published:** 2017-09-27

**Authors:** Chao Zhang, Pengcheng Zhang, Weizhan Zhang

**Affiliations:** School of Electronics and Information Engineering, Xi’an Jiaotong University, Xi’an 710049, China; zhangpengcheng@stu.xjtu.edu.cn (P.Z.); zhangwzh@mail.xjtu.edu.cn (W.Z.)

**Keywords:** wireless energy transfer, energy harvesting, cooperative communication, cluster, Markov chain

## Abstract

A wireless-powered sensor network (WPSN) consisting of one hybrid access point (HAP), a near cluster and the corresponding far cluster is investigated in this paper. These sensors are wireless-powered and they transmit information by consuming the harvested energy from signal ejected by the HAP. Sensors are able to harvest energy as well as store the harvested energy. We propose that if sensors in near cluster do not have their own information to transmit, acting as relays, they can help the sensors in a far cluster to forward information to the HAP in an amplify-and-forward (AF) manner. We use a finite Markov chain to model the dynamic variation process of the relay battery, and give a general analyzing model for WPSN with cluster cooperation. Though the model, we deduce the closed-form expression for the outage probability as the metric of this network. Finally, simulation results validate the start point of designing this paper and correctness of theoretical analysis and show how parameters have an effect on system performance. Moreover, it is also known that the outage probability of sensors in far cluster can be drastically reduced without sacrificing the performance of sensors in near cluster if the transmit power of HAP is fairly high. Furthermore, in the aspect of outage performance of far cluster, the proposed scheme significantly outperforms the direct transmission scheme without cooperation.

## 1. Introduction

Radio-Frequency (RF) based wireless energy transfer technique has recently became a newly emerging technology to charge the energy-constrained wireless networks [1,2,3,4]. As a result, wireless devices can harvest energy from RF signals to transmit information. Recently, there have been a lot of research work on a wireless-powered communication network (WPCN) [5]. Simultaneous wireless information and power transfer (SWIPT) network has been studied in [6], and optimal time allocation strategies to maximize sum-throughput and common-throughput of the WPCN have been proposed in [7]. Because of the severe loss of broadcast propagation, the authors in [8,9,10] come up with energy beamforming and directional antenna to improve energy efficiency. Power Beacon (PB) assisted WPCN is first proposed in [11]. In such networks, PBs are deployed in a cellular network as dedicated energy source to charge wireless terminals. Authors in [12] investigate outage probability of typical sensor in wireless powered sensor networks, in which PBs and distributed antennas are all randomly deployed, and deduce the closed-form expression when the path-loss exponent is 4.

Recently, terminals in WPCN are usually assumed to transmit information by exclusively consuming all the harvested energy during a transmission block. Because of the severe path loss of broadcast propagation, terminals could only harvest little energy during such little time. In addition, these WPCN systems might then cause poor performance. Thus, equipping terminals in WPCN with energy storage unit is of great urgency [13,14,15,16], so that terminals can accumulate enough energy before transmit information without waster of energy. In [17], Krikidis et al. studies a cooperative network with a single wireless-powered relay, where authors first use a finite Markov chain to model the dynamic variation process of the relay battery. The performance of the cooperative network by switching the relay operation between energy harvesting and information transmission just according to whether the relay battery level exceeds the predefined value is analyzed in [18]. Multi-relay wireless-powered cooperative communication network are analyzed in [19,20,21], where authors consider the energy accumulation of the relay battery and propose various relay section schemes. Very recently, researchers in [22,23] consider that the source can transmit information to destination directly, so that relay can assist information transmission opportunistic when relay accumulates enough harvested energy. A wireless-powered two-way relay network is investigated in [24], in which the wireless-powered relay harvests energy from RF signals ejected by two sources and help the two sources exchanging information. The idea of using wireless-powered relay for device-to device (D2D) network is proposed in [25], where user equipment harvests energy from the access point and use the accumulated energy for D2D communication by acting as a relay. Besides, in [26] the concept of wireless powered terminal in the K-tier heterogeneous network is addressed.

Wireless sensor networks have attracted more and more attention because of their low cost and wide applications. However, energy balance of the deployed sensor nodes is still an urgent issue, which concerns the lifetime of wireless sensor networks. In [27], unequal clustering is proposed to efficiently and uniformly utilize the limited energy of the cluster head nodes to increase network lifetime. Lifetime maximizing problem in sink-based wireless sensor networks is studied [28,29,30]. Based on the ant colony optimization, an optimal-distance-based transmission strategy to achieve network lifetime maximization is proposed in [28]. The uneven energy depletion phenomenon is theoretically analyzed in [29], and authors also give guidelines of optimal sizes of coronas when designing sink-based wireless sensor networks. By using corona-based network division and mixed-routing strategy, authors in [30] design an energy-balanced data gathering protocol to solve unbalanced energy consumption.

Applying the wireless energy transfer into wireless sensor networks, a dedicated energy harvesting relay for relaying information in wireless-powered cooperative communication networks (WPCCN) is studied in [31,32]. However, the node in the wireless sensor network can play roles of both source and relay in practice. As all sensors need to transmit their own data to the sink through multi-hop communication, the sensor which is close to the hybrid access point (HAP) can act as a relay for forwarding received data from the far sensors to the HAP. On the other hand, the sensors close to the HAP could harvest more energy than the ones far from the HAP. Therefore, cooperation between wireless-powered sensors may improve the performance of wireless-powered sensor networks. However, if near sensors cooperate with far sensors too intensively, this would lead to even lower battery status and cause worse system performance. Thus, the deployment of sensor cooperation in wireless-powered sensor networks is an important issue.

In this paper, we investigate a wireless-powered sensor network (WPSN) consisting of one HAP, a near sensor cluster and the corresponding far sensor cluster. We consider that each sensor has ability to harvest energy and store energy. We propose that if sensors in near cluster don’t have their own information to transmit, acting as relays, they can help the sensors in far cluster to forward information to the HAP in an Amplify-and-Forward (AF) manner. Furthermore, only when sensors in near cluster and sensors in far cluster exceed their energy threshold, respectively, could cooperation transmission be activated. Otherwise, the sensor in far cluster transmits its own data to the HAP directly. Using a finite Markov chain to model the dynamic variation process of the sensor battery, we deduce the outage probability of sensors in far cluster and in near cluster respectively. Simulation results validate the start point of designing this paper and correctness of theoretical analysis and show how parameters have effect on system performance.

## 2. System Model

**Figure 1:**
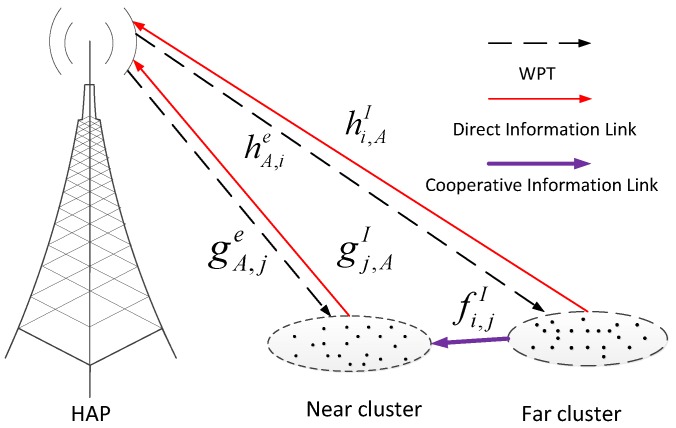
System model.

We consider a wireless-powered sensor network (WPSN), where the hybrid access point (HAP) transfers energy to all clustered sensors and these sensors perform information transmission (IT) to the HAP in the uplink. $P_{0}$ denotes the transmit power of the HAP and these sensors with close geographical positions are merged into one cluster. Since sensors in one cluster approach each other very closely, we assume the sensors in the cluster have the same distance from HAP and it is named cluster distance. As depicted in Figure 1, we consider two clusters with cluster distances *r* and *R*, where r<R. The clusters with distances *r* and *R* are called near cluster and far cluster, respectively. Generally speaking, the near cluster can harvest more energy and consume less energy for a reliable information transmission with the same target rate than the far cluster. To overcome this problem, we consider a two-hop cluster cooperative transmission scheme, where the near cluster is assigned to the far cluster, which means the near cluster tries to help the far cluster cooperatively transmit information to the HAP. It is also assumed that all sensors work in the half-duplex mode.

### 2.1. Channel Model

Let $h_{i,A}^{I}$ and $g_{j,A}^{I}$ denotes the channel power gain between the *i*-th sensor in far cluster and the HAP and that between the *j*-th sensor in near cluster and the HAP, respectively. In addition $f_{i,j}^{I}$ denote the channel power gain between the *i*-th sensor in far cluster and the *j*-th sensor in its corresponding near cluster. $h_{A,i}^{e}$ and $g_{A,j}^{e}$ represent the channel power gain between the HAP and the *i*-th sensor in far cluster and that between the HAP and the *j*-th sensor in near cluster, respectively. In addition, the channel power gains from the same cluster (or HAP) are assumed to follow independently and identically distributed (i.i.d.) exponential distribution. Herein, let α denotes the path-loss exponent of downlink WPT and β denotes the path-loss exponent of uplink information transmission (IT). All channel power gains remain constant during each transmission block, denoted by *T*, and the value is independent from block to block.

### 2.2. Cluster Cooperation Protocol

During a transmission block, each sensor sends its own data with probability *p* by using the frequency division multiple access (FDMA) scheme. When the sensor in near cluster has no data to send, it can help the sensor in the corresponding far cluster by acting as a relay for forwarding received data from the corresponding sensor to the HAP in the Amplify-and-Forward (AF) manner. In this paper, we employ a random relay matching protocol in order to reduce the system implementation complexity. In future work, more powerful relay matching protocols with more complexity can be proposed by utilizing battery status and channel gain of these sensors [33]. Before describing the operation of sensor in this network, we make the following assumptions:

1. Each sensor owns an energy receiver and an information processing module. Sensors can flexibly switch their operation between energy harvesting and information processing but can’t operate the two modules simultaneously [31].
2. Each sensor is equipped with a rechargeable battery so that sensors can store the harvested energy. Each sensor has the ability to send its own information only when the residual energy in its battery exceeds the predefined threshold $B_{a}$. In this case, we call the sensor works in active mode. On the contrary, the sensor is in inactive mode. In addition, a sensor in near cluster can act as a relay when its battery level exceeds the predefined energy threshold $B_{r}$, and we call the sensor is in relaying mode. Generally speaking, $B_{r}$ is no less than $B_{a}$ in practice.
3. Compared with energy expenditure of IT, energy expenditure of signal processing at the sensor is negligible. Surveys [3,31,32] have introduced this assumption.
4. Perfect network-level synchronization between these sensors and HAP is assumed like most of the literature [17,18,19,20,21,22,23,24,25,26].

In the wireless-powered sensor network, one transmission block *T* is divided into two phases: Energy Harvesting (EH) phase and IT phase. In the EH phase, i.e., the downlink WPT phase, all sensors need to harvest energy from the RF signal radiated by the HAP. Define the duration of the EH phase as $T_{e}=\psi T$ and the duration of IT phase as $T_{d}=\left(1-\psi \right)T$, where 0<ψ<1. For the convenience in analyzing, we suppose that IT phase is followed by the EH phase and each sensor has the initial energy supply in its battery. Due to the quite low complexity, the time for relay selection and matching is negligible as compared with the whole transmission block.

Let the binary-valued variable *X* denote the transmitting status variable of the sensor, and X=1 means the sensor should transmit its data and X=0 means there is no data to be transmitted. Similarly, let binary-valued variable $Y_{1}$ denote the indicator at the sensor in far cluster, where $Y_{1}=0$ represents the residual energy at the sensor in far cluster is short of $B_{a}$ and otherwise $Y_{1}=1$. In addition, binary-valued variables $Y_{2}$ and $Y_{3}$ denote the indicators at the sensor in near cluster. $Y_{2}=0$ indicates the residual energy at the sensor is short of $B_{a}$ and otherwise $Y_{2}=1$. $Y_{3}$ equals to 0 stands for that the available energy at the sensor is short of $B_{r}$ and otherwise there is $Y_{3}=1$. The relay request indicator at the sensor in far cluster is denoted as $Z_{1}$, where we have $Z_{1}=1$ if the sensor requests a relay in near cluster successfully and otherwise $Z_{1}=0$. The binary-valued variable $Z_{2}$ denote the relay request indicator at the sensor in near cluster, which equals to 1 if the sensor is requested as a relay and becomes 0 otherwise. Since all sensors only harvest energy during the EH phase, we just need to study the sensor operation in the IT phase. Next, we intend to describe the scheme in detail.

#### 2.2.1. Working Modes in Far Cluster

According to our cluster cooperation protocol, the sensor in far cluster works in the following modes:

- Mode I ($Y_{1}=0$): In this mode, the sensor is in inactive mode since the battery level is under $B_{a}$.
- Mode II (X=0 and $Y_{1}=1$): Although there exists enough energy for the sensor transmitting information, there is no data to be transmitted. Thus, the sensor behaves like in Mode I.
- Mode III ($X=1,\;Y_{1}=1$ and $Z_{1}=0$): In this mode, the sensor has data to transmit but requests a relay unsuccessfully, so that the sensor transmits data to the HAP directly by consuming $B_{a}$ amount of energy from its battery.
- Mode IV ($X=1,\;Y_{1}=1$ and $Z_{1}=1$): The sensor has the ability to transmit its data since the battery level exceeds $B_{a}$ and the sensor requests a relay successfully. When the relay is in active mode, IT phase $T_{d}$ is divided into two equal time slots with duration $T_{d}/2$. The sensor transmits its information to the HAP and the matched relay in the first time slot. In the second time slot, the relay forwards the amplified signal to the HAP by consuming $B_{a}$ amount of energy from its battery. If the relay is inactive at the same time, it has to remain silent and does nothing during the IT phase.

We will analyze the operation of sensors in the far cluster and the received signal-to-noise ratio (SNR) at the HAP for the above four modes. To simplify the notations, let $h_{I}$ and $g_{I}$ denote the channel power gain between the typical sensor in the far cluster and the HAP and that between the typical sensor in the near cluster and the HAP, respectively. Let $f_{I}$ denote the channel power gain between the typical sensor in the far cluster and the typical sensor in its corresponding near cluster. Furthermore, the typical sensor in the near cluster would be the relay of the typical sensor in the far cluster. Similarly, $h_{e}$ and $g_{e}$ denote the channel power gain between the HAP and typical sensor in the far cluster and that between the HAP and typical sensor in the near cluster, respectively. Without loss of generality, we consider an unit transmission block, i.e., T=1, hereafter.

In Mode I, the sensor is in inactive mode since the battery level is under $B_{a}$, so that it doesn’t have the ability to transmit information. Even if the sensor has data to transmit, the received SNR at the HAP is also zero. In Mode II, the sensor has enough energy but has no data to be transmitted, so it just harvests energy during EH phase and does nothing during IT phase. In Mode III, the sensor has enough energy and can send data, but requests a relay unsuccessfully. Therefore, the sensor transmits data to the HAP directly in the whole transmission block by consuming $B_{a}$ amount of energy from its battery. The received SNR at the HAP is

(1) $$\gamma_{D}^{f}=\frac{B_{a}h_{I}}{R^{\beta }N_{0}\left(1-\psi \right)},$$

where $N_{0}$ is the additive Gaussian white noise power. In Mode IV, the sensor requests a relay successfully. If the relay works in active mode, the received SNR at the corresponding relay in the first time slot is

(2) $$\gamma_{r_{1}}=\frac{2B_{a}f_{I}}{d_{R}^{\beta }N_{0}\left(1-\psi \right)},$$

where $d_{R}$ is the distance from the far cluster to the near cluster, and the received SNR at the HAP is

(3) $$\gamma_{d}=\frac{2B_{a}h_{I}}{R^{\beta }N_{0}\left(1-\psi \right)}.$$

In the second time slot, the relay transmits the received data to the HAP in AF manner at the expense of $B_{a}$ amount of energy. The received SNR at the HAP is

(4) $$\gamma_{r_{2}}=\frac{2B_{a}g_{I}}{r^{\beta }N_{0}\left(1-\psi \right)}.$$

Let $\gamma_{r}$ represent the received SNR via a relay. For the AF relay, $\gamma_{r}$ is

(5) $$\gamma_{r}=\frac{\gamma_{r_{1}}\gamma_{r_{2}}}{1+\gamma_{r_{1}}+\gamma_{r_{2}}}.$$

By using the maximum ratio combining (MRC) technique, the received SNR at the HAP through the whole transmission block is expressed as

(6) $$\gamma_{AF}=\gamma_{d}+\gamma_{r}.$$

When the relay is inactive, the received SNR is the same as the Mode III.

Since the receiver noise power is too small to harvest, we always ignore the energy harvested from the noise. Then, the harvested energy at the sensor is given by

(7) $$E_{H_{1}}=\psi \eta P_{0}h_{e}R^{-\alpha },$$

where η∈(0,1] denote the RF-to-Direct Current (DC) conversion efficiency.

#### 2.2.2. Working Modes in Near Cluster

Similarly, the sensor in near cluster operates in the following six modes:

- Mode I ($Y_{2}=0$): In this mode, the sensor is in inactive mode since the battery level is under $B_{a}$.
- Mode II (X=1, and $Y_{2}=1$): The sensor is in active mode and has data to transmit, so that the sensor transmits data to the HAP directly by consuming $B_{a}$ amount of energy from its battery.
- Mode III ($X=0,\;Y_{2}=1$ and $Y_{3}=0$ ): Here, the sensor is in active mode and has no data to transmit. Furthermore, the energy in the battery is less than $B_{r}$, so the sensor doesn’t have the ability to act as a relay.
- Mode IV ($X=0,\;Y_{3}=1$ and $Z_{2}=0$): In this mode, $Y_{3}=1$ indicates the available energy at the sensor exceeds $B_{r}$, but the sensor neither has data to transmit nor is requested as a relay.
- Mode V ($X=0,\;Y_{3}=1,\;Z_{2}=1$ and $Y_{1}=1$): Here, the available energy at the sensor exceeds $B_{r}$ and the sensor is requested as a relay. In addition, the corresponding sensor in far cluster is in active mode, so the IT time is divided into two equal time slots with duration $T_{d}/2$. The sensor receives the signal broadcasted by the corresponding sensor in far cluster in the first time slot. In addition in the second time slot, the sensor forwards the amplified signal to the HAP by consuming $B_{a}$ amount of energy from its battery.
- Mode VI ($X=0,\;Y_{3}=1,\;Z_{2}=1$ and $Y_{1}=0$): The only difference from mode V is that the corresponding sensor in far cluster is in inactive mode, the sensor in near cluster does nothing during IT phase.

IT phase $T_{d}$ is divided into two equal time slots. The sensor transmits its information to the HAP and the matched relay in the first time slot.

In this part, we only consider the received SNR at the HAP when the sensor in near cluster transmits its own information. According to the above analysis, the sensor in near cluster might transmit its own data in Modes I and II. In Mode I, the sensor is in inactive mode since the battery level is under $B_{a}$, so that it doesn’t have ability to transmit information. If the sensor has data to transmit, the received SNR at the HAP is zero. In Mode II, the sensor is in active mode and sends data, so the sensor transmits data to the HAP directly in the whole transmission block by consuming $B_{r}$ amount of energy. The SNR at the HAP is

(8) $$\gamma_{D}^{n}=\frac{B_{a}g_{I}}{r^{\beta }N_{0}\left(1-\psi \right)}.$$

In Mode III, the energy in the sensor battery exceeds $B_{a}$ and is less than $B_{r}$; furthermore, the sensor has no data to transmit. In Mode IV, the sensor has enough energy but neither sends data nor is requested as a relay, so it harvests energy in EH phase and do nothing during IT phase. In Mode V, the sensor is requested as a relay for helping the corresponding sensor in far cluster to transmit information and the corresponding sensor in far cluster is in active mode, so that the sensor transmits the received data to the HAP at the expense of $B_{a}$ amount of energy. In Mode VI, the corresponding sensor in far cluster is in inactive mode, so there is no need for the sensor in near cluster acting as a relay. The sensor behaves like Modes IV and V.

Like the sensor in far cluster, the harvested energy at the sensor during the EH phase is expressed as

(9) $$E_{H_{2}}=\psi \eta P_{0}g_{e}r^{-\alpha }.$$

## 3. Performance Analysis

We will use outage probability as the metric to evaluate network performance over Rayleigh fading channels. Before analyzing, we need to obtain two important conditional probabilities in the following context.

Due to the system model, the probability that the far cluster has data to be transmitted to HAP can be expressed as $1-{\left(1-p\right)}^{N}$. Considering the random relay matching protocol, the probability that the sensor in far cluster requests a relay in the corresponding near cluster successfully while has data to be transmitted to HAP is ∑k=1N∑l=0MNkpk(1−p)N−kMl(1−p)lpM−lminlk,1, where Nk stands for the combinatorial number of choosing k elements from the set with N elements. Note that $\min\left\{\frac{l}{k},1\right\}$ means that if the number of available sensor in near cluster is larger than the number of sensors with data to be transmitted in far cluster, the requirements from far cluster must be satisfied by near cluster. Otherwise, the sensor with data to be transmitted in far cluster has to randomly choose one of available sensors in near cluster as the relay. Then, the probability that the sensor in far cluster requests a relay in the corresponding near cluster successfully under the condition that it has data to be transmitted to HAP is

(10) Pr=∑k=1N∑l=0MNkpk(1−p)N−kMl(1−p)lpM−lminlk,11−1−pN−1,

where *M* and *N* denote the number of sensors in near cluster and the corresponding far cluster, respectively. Similarly, the conditional probability that the sensor in near cluster is chosen as a relay if it has no data for HAP is

(11) q=∑k=0N∑l=1MNkpk(1−p)N−kMl(1−p)lpM−lminkl,11−pM−1.

To figure out the dynamic behavior of the stored energy in the sensor node, the approach of dividing the battery into discrete levels and using Markov Chain to describe the behavior of energy state is usually employed to analyze the system performance, e.g., [19,20,21,22,23,24,25,26,34,35]. Thus, we also use a finite Markov chain to model the dynamic variation process of the relay battery.

### 3.1. Markov Chain Model of the Sensor in the Far Cluster

**Figure 2:**
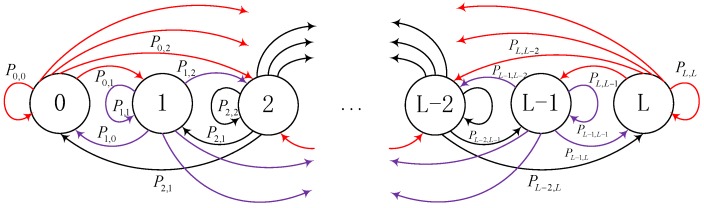
State transition probability of Markov chain.

We assume that all sensors are equipped with a rechargeable battery and its finite capacity is *C*. In addition, we then divide the battery level into *L* discrete levels. Let $\nu_{i}=iC/L,i\in \left\{0,1,2,\;\ldots ,L\right\}$ denote the sensor battery being the *i*th discrete energy level, and the corresponding state is defined as $S_{i}$ . As showed in Figure 2, $P_{i,j}$ denotes the state transition probability $P\{S_{i}\to S_{j}\}$. The effective harvested energy at the sensor in far cluster is $\nu_{H_{1}}≜\nu_{i}$, where $i=\arg\;\max_{j\in \left\{0,1,\;\cdots ,L\right\}}\{\nu_{j}$:$\nu_{j}\leq E_{H_{1}}\}$. In addition the energy expenditure for information transmission is $\nu_{T_{1}}≜\nu_{i}$, where $i=\arg\;\min_{j\in \left\{0,1,\;\cdots ,L\right\}}\{\nu_{j}$:$\nu_{j}\geq B_{a}\}$. Let $P_{i,j}^{f}$ denote the state transition probability of the sensor in far cluster and we introduce the state transition probability of sensor in far cluster:

(1) $S_{0}\to S_{0}$: Battery level transforms from 0-th state to 0-th state. This is because the sensor is in inactive mode and the harvested energy is less that $\frac{C}{L}$. In this case, the state transition probability is
  (12) $$P_{0,0}^{f}=P\left(E_{H_{1}}<\frac{C}{L}\right)=F_{H_{e}}\left(\frac{CR^{\alpha }}{\psi \eta P_{0}L}\right),$$
  where $F_{H_{e}}\left(\cdot \right)$ is the cumulative distribution function (CDF) of exponential random variable $h_{e}$ with mean value $\bar{h_{e}}$ as $F_{H_{e}}\left(x\right)=1-exp\left(-\frac{x}{\bar{h_{e}}}\right)$.
(2) $S_{0}\to S_{i}$ (0<i<L): Battery level transforms from 0-th state to *i*-th state. The sensor is in inactive mode and the effective amount of harvested energy is $\frac{iC}{L}$. In this case, the corresponding state transition probability is
  (13) $$\begin{array}{c} P_{0,i}^{f}=P\left(\frac{iC}{L}\leq E_{H_{1}}<\frac{(i+1)C}{L}\right)=F_{H_{e}}\left(\frac{\left(i+1\right)CR^{\alpha }}{\psi \eta P_{0}L}\right)-F_{H_{e}}\left(\frac{iCR^{\alpha }}{\psi \eta P_{0}L}\right). \end{array}$$
(3) $S_{0}\to S_{L}$: Battery level transforms from 0-th state to *L*-th state. The sensor works in inactive mode and the harvested energy is more than the battery capacity with the corresponding state transition probability:
  (14) $$P_{0,L}^{f}=P\left(E_{H_{1}}\geq C\right)=1-F_{H_{e}}\left(\frac{CR^{\alpha }}{\psi \eta P_{0}}\right).$$
(4) $S_{i}\to S_{i}$ (0<i<L): Battery level transforms from *i*-th state to *i*-th state, which means that the sensor works doesn’t transmit information or works in inactive mode with the harvested energy being less than $\frac{C}{L}$. Another possibility is the sensor is in active mode and transmits information with $\nu_{T_{1}}$ effective harvested energy. In addition, we can use $XY_{1}=0$ to represent the sensor works in Mode I or Mode II without consuming any energy, and use $XY_{1}=1$ to represent the sensor operates in Mode III or Mode IV with consuming $B_{1}$ amount of energy. In this case, the state transition probability is
  (15) $$\begin{array}{cc} P_{i,i}^{f} & =P\left\{\left[\left(XY_{1}=0\right)\cap \left(E_{H_{1}}<\frac{C}{L}\right)\right]\cup \left[\left(XY_{1}=1\right)\cap \left(\frac{T_{1}C}{L}\leq E_{H_{1}}<\frac{(T_{1}+1)C}{L}\right)\right]\right\},\\ & =\left\{\begin{array}{cc} F_{H_{e}}\left(\frac{CR^{\alpha }}{\psi \eta P_{0}L}\right), & \mathrm{if}\;i<T_{1},\\ \left(1-p\right)F_{H_{e}}\left(\frac{CR^{\alpha }}{\psi \eta P_{0}L}\right)+p\left[F_{H_{e}}\left(\frac{\left(T_{1}+1\right)CR^{\alpha }}{\psi \eta P_{0}L}\right)-F_{H_{e}}\left(\frac{T_{1}CR^{\alpha }}{\psi \eta P_{0}L}\right)\right], & \mathrm{if}\;i\geq T_{1}. \end{array}\right. \end{array}$$
(5) $S_{i}\to S_{j}$ (0<i<j<L): Battery level transforms from *i*-th state to *j*-th state. Similar to the above case, the effective harvested energy is $\frac{(j-i)C}{L}$ or $\frac{\left(j-i+T_{1}\right)C}{L}$, we can obtain the state transition probability:
  (16) $$\begin{array}{cc} P_{i,j}^{f} & =P\left\{\left[\left(XY_{1}=0\right)\cap \left(\frac{(j-i)C}{L}\leq E_{H_{1}}<\frac{(j-i+1)C}{L}\right)\right],\right.\\ & \left.\;\;\;\cup \left[\left(XY_{1}=1\right)\cap \left(\frac{(j-i+T_{1})C}{L}\leq E_{H_{1}}<\frac{(j-i+T_{1}+1)C}{L}\right)\right]\right\},\\ & =\left\{\begin{array}{cc} F_{H_{e}}\left(\frac{\left(j-i+1\right)CR^{\alpha }}{\psi \eta P_{0}L}\right)-F_{H_{e}}\left(\frac{\left(j-i\right)CR^{\alpha }}{\psi \eta P_{0}L}\right), & \mathrm{if}\;i<T_{1},\\ \left(1-p\right)\left[F_{H_{e}}\left(\frac{\left(j-i+1\right)CR^{\alpha }}{\psi \eta P_{0}L}\right)-F_{H_{e}}\left(\frac{\left(j-i\right)CR^{\alpha }}{\psi \eta P_{0}L}\right)\right]+\\ p\left[F_{H_{e}}\left(\frac{\left(j-i+T_{1}+1\right)CR^{\alpha }}{\psi \eta P_{0}L}\right)-F_{H_{e}}\left(\frac{\left(j-i+T_{1}\right)CR^{\alpha }}{\psi \eta P_{0}L}\right)\right], & \mathrm{if}\;i\geq T_{1}. \end{array}\right. \end{array}$$
(6) $S_{i}\to S_{L}$ (0<i<L): Battery level transforms from *i*-th state to *L*-th state. The effective harvested energy is more than $\frac{\left(L-i\right)C}{L}$ if the sensor operates in inactive mode or in active without transmitting information, or is more than $\frac{\left(L-i+T_{1}\right)C}{L}$ if the sensor is in active mode with transmitting information. In this case, the state transition probability is
  (17) $$\begin{array}{cc} P_{i,L}^{f} & =P\left\{\left[\left(XY_{1}=0\right)\cap \left(E_{H_{1}}\geq \frac{(L-i)C}{L}\right)\right]\cup \left[\left(XY_{1}=1\right)\cap \left(E_{H_{1}}\geq \frac{(L-i+T_{1})C}{L}\right)\right]\right\},\\ & =\left\{\begin{array}{cc} 1-F_{H_{e}}\left(\frac{\left(L-i\right)CR^{\alpha }}{\psi \eta P_{0}L}\right), & \mathrm{if}\;i<T_{1},\\ \left(1-p\right)\left[1-F_{H_{e}}\left(\frac{\left(L-i\right)CR^{\alpha }}{\psi \eta P_{0}L}\right)\right]+p\left[1-F_{H_{e}}\left(\frac{\left(L-i+T_{1}\right)CR^{\alpha }}{\psi \eta P_{0}L}\right)\right], & \mathrm{if}\;i\geq T_{1}\;\mathrm{and}\;L-i\geq T. \end{array}\right. \end{array}$$
(7) $S_{L}\to S_{L}$: Battery level transforms from *L*-th state to *L*-th state. The sensor must be in active mode. Thus, if the sensor doesn’t consume any energy, the battery level must remain full. In addition, if the sensor consumes $B_{a}$ amount of energy, the effective harvest energy being greater than $\nu_{T_{1}}$ also meets the condition. In this case, the corresponding state transition probability is
  (18) $$\begin{array}{c} P_{L,L}^{f}=P\left\{\left[x=0\right]\cup \left[\left(X=1\right)\cap \left(E_{H_{1}}\geq \frac{T_{1}C}{L}\right)\right]\right\}=1-p+p\left[1-F_{H_{e}}\left(\frac{T_{1}CR^{\alpha }}{\psi \eta P_{0}L}\right)\right]. \end{array}$$
(8) $S_{j}\to S_{i}$ (0<i<j≤L): Battery level transforms from *j*-th state to *i*-th state. According to the operation of the sensor in far cluster, the battery level is decreased when the sensor works in active mode with transmitting information. In this case, the state transition probability is
  (19) $$\begin{array}{c} P_{j,i}^{f}=P\left\{\left(Y_{1}=1\right)\cap \left(j-i\leq T_{1}\right)\right\}=\left\{\begin{array}{cc} p\left[F_{H_{e}}\left(\frac{\left(i-j+T_{1}+1\right)CR^{\alpha }}{\psi \eta P_{0}L}\right)-F_{H_{e}}\left(\frac{\left(i-j+T_{1}\right)CR^{\alpha }}{\psi \eta P_{0}L}\right)\right], & \mathrm{if}\;j-i\leq T_{1},\\ 0, & \mathrm{if}\;j-i\neq T_{1}. \end{array}\right. \end{array}$$

Let $P_{1}={\left[P_{i,j}^{f}\right]}_{\left(L+1)\times (L+1\right)}$ represent the state transition matrix of the sensor in far cluster. We can easily conclude that $P_{1}$ is irreducible and row stochastic. Hence, let $\pi_{1}=\left(\pi_{10},\pi_{11},\;\ldots ,\pi_{1L}\right)$ denote the steady state distribution of the sensor in far cluster and it can be expressed as

(20) $$\pi_{1}={\left(P_{1}^{T}-I+A\right)}^{-1}a,$$

where $P_{1}^{T}$ is the transpose matrix of $P_{1}$, I is the identity matrix, $A=[A_{i,j}]$ and $A_{i,j}=1,\forall i,j$, and $a={\left(1,1,\;\ldots ,1\right)}^{T}$. Let $P_{E_{1}}$ denote the probability of the sensor in far cluster being in active mode, which is

(21) $$P_{E_{1}}=\Sigma_{i=T_{1}}^{L}\pi_{1i}.$$

Note that $P_{E_{1}}$ would be used in the following analysis.

### 3.2. Markov Chain Model of the Sensor in Near Cluster

Similar to the above deduction, the discrete harvested energy $\nu_{H_{2}}$ at the sensor in far cluster should be $\nu_{H_{2}}≜\nu_{i}$, where $i=\arg\;\max_{j\in \left\{0,1,\;\cdots ,L\right\}}\{\nu_{j}$:$\nu_{j}\leq E_{H_{2}}\}$. In addition, the corresponding battery level needed by the sensor in near cluster for information relaying is $\nu_{T_{2}}≜\nu_{i}$, where $i=\arg\;\min_{j\in \left\{0,1,\;\cdots ,L\right\}}\{\nu_{j}$:$\nu_{j}\geq B_{2}\}$. Let $P_{i,j}^{n}$ denote the state transition probability from $S_{i}$ to $S_{j}$ of the sensor in near cluster, and we can get the following deduction:

(1) $S_{0}\to S_{0}$: Battery level transforms from 0-th state to 0-th state. Just like the above section, the sensor operates in inactive mode and the harvested energy is less that $\frac{C}{L}$. In this case, the state transition probability is
  (22) $$P_{0,0}^{n}=P\left(E_{H_{2}}<\frac{C}{L}\right)=F_{G_{e}}\left(\frac{Cr^{\alpha }}{\psi \eta P_{0}L}\right),$$
  where $F_{G_{e}}\left(\cdot \right)$ is the CDF of exponential random variable $g_{e}$ with mean value $\bar{g_{e}}$ as $F_{G_{e}}\left(x\right)=1-exp\left(-\frac{x}{\bar{g_{e}}}\right)$.
(2) $S_{0}\to S_{i}$ (0<i<L): Battery level transforms from 0-th state to *i*-th state. The sensor is in inactive mode and the effective amount of harvested energy is $\frac{iC}{L}$. In this case, the corresponding state transition probability is
  (23) $$\begin{array}{c} P_{0,i}^{n}=P\left(\frac{iC}{L}\leq E_{H_{2}}<\frac{(i+1)C}{L}\right)=F_{G_{e}}\left(\frac{\left(i+1\right)Cr^{\alpha }}{\psi \eta P_{0}L}\right)-F_{G_{e}}\left(\frac{iCr^{\alpha }}{\psi \eta P_{0}L}\right). \end{array}$$
(3) $S_{0}\to S_{L}$: Battery level transforms from 0-th state to *L*-th state. The sensor works in inactive mode and $E_{H_{2}}\geq C$. In this case, the state transition probability is
  (24) $$P_{0,L}^{n}=P\left(E_{H_{2}}\geq C\right)=1-F_{G_{e}}\left(\frac{Cr^{\alpha }}{\psi \eta P_{0}}\right).$$
(4) $S_{i}\to S_{i}$ (0<i<L): Battery level transforms from *i*-th state to *i*-th state. The sensor works in inactive mode or in active mode without transmitting information or in relaying mode without relaying information with zero effective harvested energy, or the sensor is in active mode with transmitting information or in relaying mode with relaying information with $\nu_{T_{1}}$ effective harvested energy. We can use $\left(XY_{2}+Y_{3}Z_{2}Y_{1}=0\right)$ to represent the sensor operates in Mode I, Mode III, Mode IV, or Mode VI without consuming any energy, and use $\left(XY_{2}+Y_{3}Z_{2}Y_{1}=1\right)$ to represent the sensor operates in Mode II or Mode V with consuming $B_{1}$ amount of energy. For simplicity, we use $P_{u}$ to denote the probability of the sensor in near cluster consuming energy when the residual energy exceeds $B_{2}$, and it can be expressed as $P_{u}=p+\left(1-p\right)qP_{E_{1}}$. Thus, the state transition probability is
  (25) $$\begin{array}{cc} P_{i,i}^{n} & =P\left\{\left[\left(XY_{2}+Y_{3}Z_{2}Y_{1}=0\right)\cap \left(E_{H_{2}}<\frac{C}{L}\right)\right]\cup \left[\left(XY_{2}+Y_{3}Z_{2}Y_{1}=1\right)\cap \left(\frac{T_{1}C}{L}\leq E_{H_{2}}<\frac{\left(T_{1}+1\right)C}{L}\right)\right]\right\},\\ & =\left\{\begin{array}{cc} F_{G_{e}}\left(\frac{Cr^{\alpha }}{\psi \eta P_{0}L}\right), & \mathrm{if}\;i<T_{1},\\ \left(1-p\right)F_{G_{e}}\left(\frac{Cr^{\alpha }}{\psi \eta P_{0}L}\right)+p\left[F_{G_{e}}\left(\frac{\left(T_{1}+1\right)Cr^{\alpha }}{\psi \eta P_{0}L}\right)-F_{G_{e}}\left(\frac{T_{1}Cr^{\alpha }}{\psi \eta P_{0}L}\right)\right], & \mathrm{if}\;T_{1}\leq i<T_{2},\\ \left(1-P_{u}\right)F_{G_{e}}\left(\frac{Cr^{\alpha }}{\psi \eta P_{0}L}\right)+P_{u}\left[F_{G_{e}}\left(\frac{\left(T_{1}+1\right)Cr^{\alpha }}{\psi \eta P_{0}L}\right)-F_{G_{e}}\left(\frac{T_{1}Cr^{\alpha }}{\psi \eta P_{0}L}\right)\right], & \mathrm{if}\;i\geq T_{2}. \end{array}\right. \end{array}$$
(5) $S_{i}\to S_{j}$ (0<i<j<L): Battery level transforms from *i*-th state to *j*-th state. Similar to the above case, the effective harvested energy is $\frac{(j-i)C}{L}$ or $\frac{\left(j-i+T_{1}\right)C}{L}$ with the state transition probability:
  (26) $$\begin{array}{cc} P_{i,j}^{n} & =P\left\{\left[\left(XY_{2}+Y_{3}Z_{2}Y_{1}=0\right)\cap \left(\frac{(j-i)C}{L}\leq E_{H_{2}}<\frac{(j-i+1)C}{L}\right)\right],\right.\\ & \left.\;\;\;\cup \left[\left(XY_{2}+Y_{3}Z_{2}Y_{1}=1\right)\cap \left(\frac{(j-i+T_{1})C}{L}\leq E_{H_{2}}<\frac{(j-i+T_{1}+1)C}{L}\right)\right]\right\},\\ & =\left\{\begin{array}{cc} F_{G_{e}}\left(\frac{\left(j-i+1\right)Cr^{\alpha }}{\psi \eta P_{0}L}\right)-F_{G_{e}}\left(\frac{\left(j-i\right)Cr^{\alpha }}{\psi \eta P_{0}L}\right), & \mathrm{if}\;i<T_{1},\\ \left(1-p\right)\left[F_{G_{e}}\left(\frac{\left(j-i+1\right)Cr^{\alpha }}{\psi \eta P_{0}L}\right)-F_{G_{e}}\left(\frac{\left(j-i\right)Cr^{\alpha }}{\psi \eta P_{0}L}\right)\right]+\\ p\left[F_{G_{e}}\left(\frac{\left(j-i+T_{1}+1\right)Cr^{\alpha }}{\psi \eta P_{0}L}\right)-F_{G_{e}}\left(\frac{\left(j-i+T_{1}\right)Cr^{\alpha }}{\psi \eta P_{0}L}\right)\right], & \mathrm{if}\;T_{1}\leq i<T_{2},\\ \left(1-P_{u}\right)\left[F_{G_{e}}\left(\frac{\left(j-i+1\right)Cr^{\alpha }}{\psi \eta P_{0}L}\right)-F_{G_{e}}\left(\frac{\left(j-i\right)Cr^{\alpha }}{\psi \eta P_{0}L}\right)\right]+\\ P_{u}\left[F_{G_{e}}\left(\frac{\left(j-i+T_{1}+1\right)Cr^{\alpha }}{\psi \eta P_{0}L}\right)-F_{G_{e}}\left(\frac{\left(j-i+T_{1}\right)Cr^{\alpha }}{\psi \eta P_{0}L}\right)\right], & \mathrm{if}\;i\geq T_{2}. \end{array}\right. \end{array}$$
(6) $S_{i}\to S_{L}$ (0<i<L): Battery level transforms from *i*-th state to *L*-th state. Thus, the effective harvested energy is more than $\frac{\left(L-i\right)C}{L}$ if the sensor doesn’t consume any energy, or is more than $\frac{\left(L-i+T_{1}\right)C}{L}$ if the sensor consumes $\frac{T_{1}C}{L}$ amount of energy. In this case, the corresponding state transition probability is
  (27) $$\begin{array}{cc} P_{i,L}^{n} & =P\left\{\left[\left(XY_{2}+Y_{3}Z_{2}Y_{1}=0\right)\cap \left(E_{H_{2}}\geq \frac{(L-i)C}{L}\right)\right],\right.\\ & \left.\;\;\;\cup \left[\left(XY_{2}+Y_{3}Z_{2}Y_{1}=1\right)\cap \left(E_{H_{2}}\geq \frac{(L-i+T_{1})C}{L}\right)\right]\right\},\\ & =\left\{\begin{array}{cc} 1-F_{G_{e}}\left(\frac{\left(L-i\right)Cr^{\alpha }}{\psi \eta P_{0}L}\right), & \mathrm{if}\;i<T_{1},\\ \left(1-p\right)\left[1-F_{G_{e}}\left(\frac{\left(L-i\right)Cr^{\alpha }}{\psi \eta P_{0}L}\right)\right]+p\left[1-F_{G_{e}}\left(\frac{\left(L-i+T_{2}\right)Cr^{\alpha }}{\psi \eta P_{0}L}\right)\right], & \mathrm{if}\;T_{1}\leq i<T_{2},\\ \left(1-P_{u}\right)\left[1-F_{G_{e}}\left(\frac{\left(L-i\right)Cr^{\alpha }}{\psi \eta P_{0}L}\right)\right]+P_{u}\left[1-F_{G_{e}}\left(\frac{\left(L-i+T_{1}\right)Cr^{\alpha }}{\psi \eta P_{0}L}\right)\right], & \mathrm{if}\;i\geq T_{2}. \end{array}\right. \end{array}$$
(7) $S_{L}\to S_{L}$: Battery level transforms from *L*-th state to *L*-th state. The sensor must be in active mode. Thus, the battery level must remain full if the sensor doesn’t consume any energy. In addition, the effective harvest energy being greater than $\nu_{T_{1}}$ also meets the condition if the sensor consumes $B_{a}$ amount of energy. In this case, the corresponding state transition probability is
  (28) $$\begin{array}{cc} P_{L,L}^{n} & =P\left\{\left(X+Z_{2}Y_{1}=0\right)\cup \left[\left(X+Z_{2}Y_{1}=1\right)\cap \left(E_{H_{2}}\geq \frac{T_{1}C}{L}\right)\right]\right\}\\ & =\left(1-P_{u}\right)+P_{u}\left[1-F_{G_{e}}\left(\frac{T_{1}Cr^{\alpha }}{\psi \eta P_{0}L}\right)\right]. \end{array}$$
(8) $S_{j}\to S_{i}$ (0<i<j≤L): Battery level transforms from *j*-th state to *i*-th state. According to the operation of the sensor in near cluster, the battery level might be decreased when the sensor works in active mode with transmitting information or in relaying mode with relaying information. In this case, the state transition probability is
  (29) $$\begin{array}{cc} P_{j,i}^{n} & =P\left\{\left(X+Z_{2}Y_{1}=1\right)\cap \left(j-i\leq T_{1}\right)\right\},\\ & =\left\{\begin{array}{cc} p\left[F_{G_{e}}\left(\frac{\left(i-j+T_{1}+1\right)Cr^{\alpha }}{\psi \eta P_{0}L}\right)-F_{G_{e}}\left(\frac{\left(i-j+T_{1}\right)Cr^{\alpha }}{\psi \eta P_{0}L}\right)\right], & \mathrm{if}\;j-i\leq T_{1}\;and\;T_{1}\leq j<T_{2},\\ P_{u}\left[F_{G_{e}}\left(\frac{\left(i-j+T_{1}+1\right)Cr^{\alpha }}{\psi \eta P_{0}L}\right)-F_{G_{e}}\left(\frac{\left(i-j+T_{1}\right)Cr^{\alpha }}{\psi \eta P_{0}L}\right)\right], & \mathrm{if}\;j-i\leq T_{1}\;and\;j\geq T_{2},\\ 0, & \mathrm{otherwise}. \end{array}\right. \end{array}$$

Similar to the above deduction, let $P_{2}={\left[P_{i,j}^{n}\right]}_{\left(L+1)\times (L+1\right)}$ represent the state transition matrix of the sensor in near cluster, and $P_{2}$ is irreducible and also row stochastic. Let $\pi_{2}=\left(\pi_{20},\pi_{21},\;\ldots ,\pi_{2L}\right)$ denote the state transition matrix of the sensor in near cluster and it can be expressed as

(30) $$\pi_{2}={\left(P_{2}^{T}-I+A\right)}^{-1}a.$$

Similarly, let $P_{E_{2}}$ denote the probability of the sensor in near cluster being in active mode, which can be expressed as

(31) $$P_{E_{2}}=\Sigma_{i=T_{1}}^{L}\pi_{2i}.$$

### 3.3. Outage Probability Analysis

According to the above analysis, we first deduce the outage probability of the sensor in far cluster. An outage happening is that the link capacity is smaller than the transmission rate [20]. Let R(bits/Hz) denote the target transmission quantity of data for one sensor. An outage occurs in the direct link when the received SNR at the HAP is less than $\theta_{d}=2^{R/(1-\psi )}-1$, and an outage occurs through the cooperative transmission when the received SNR at the HAP is less than $\theta_{r}=2^{2R/(1-\psi )}-1$ due to the halved spectrum efficiency. Let $P_{o}^{f}\left\{I\right\}$ denote the outage event when the sensor in far cluster works in Mode I, and by this analogy. The outage probability of the sensor in far cluster can be expressed as

(32) $$P_{out}^{far}=\left(1-P_{E_{1}}\right)P_{o}^{f}\left\{I\right\}+P_{E_{1}}P_{o}^{f}\left\{II\right\}+P_{E_{1}}\left(1-P_{r}\right)P_{o}^{f}\left\{III\right\}+P_{E_{1}}P_{r}P_{o}^{f}\left\{IV\right\}.$$

In addition, it is clear that $P_{o}^{f}\left\{I\right\}=1$ and $P_{o}^{f}\left\{II\right\}=0$. For Mode III, the outage probability is

(33) $$P_{o}^{f}\left\{III\right\}=P\left(\gamma_{D}^{f}<\theta_{d}\right)=F_{H_{I}}\left(\frac{R^{\beta }N_{0}\theta_{d}\left(1-\psi \right)}{B_{a}}\right),$$

where $F_{H_{I}}\left(\cdot \right)$ denotes the CDF of exponential random variable $h_{I}$ with mean value $\bar{h_{I}}$ as $F_{H_{I}}\left(x\right)=1-exp\left(-\frac{x}{\bar{h_{I}}}\right)$. Similarly, for Mode IV, the outage probability can be characterized as

(34) $$\begin{array}{cc} P_{o}^{f}\left\{IV\right\} & =\left(1-P_{E_{3}}\right)P\left(\gamma_{D}^{f}<\theta_{d}\right)+P_{E_{3}}P\left(\gamma_{AF}<\theta_{r}\right)\\ & =\left(1-P_{E_{3}}\right)F_{H_{I}}\left(\frac{R^{\beta }N_{0}\theta_{d}\left(1-\psi \right)}{B_{a}}\right)+P_{E_{3}}\int_{0}^{\infty }\int_{0}^{\infty }F_{H_{I}}\left[\frac{\theta_{r}-\gamma_{r}}{2B_{a}}N_{0}R^{\beta }\left(1-\psi \right)\right]f_{g_{I}}\left(x\right)f_{f_{I}}dxdy, \end{array}$$

where $P_{E_{3}}$ denotes the probability of the residual energy at the sensor in the near cluster being no less than the energy threshold $B_{r}$, which can be derived as

(35) $$P_{E_{3}}=\Sigma_{i=T_{2}}^{L}\pi_{2i},$$

and $f_{g_{I}}\left(\cdot \right)$ and $f_{f_{I}}\left(\cdot \right)$ denote the probability density function (PDF) of exponential random variable $g_{I}$ and $f_{I}$ with $f_{g_{I}}\left(x\right)=\frac{1}{\bar{g_{I}}}exp\left(-\frac{x}{\bar{g_{I}}}\right)$ and $f_{f_{I}}\left(x\right)=\frac{1}{\bar{f_{I}}}exp\left(-\frac{x}{\bar{f_{I}}}\right)$. By substituting the terms derived above into Equation (32), we get the outage probability of the sensor in far cluster.

Similarly, we now infer the outage probability of the sensor in near cluster. Let $P_{o}^{n}\left\{I\right\}$ denote the outage event when the sensor in near cluster works in Mode I, and by this analogy. Due to the sensor transmitting its own data when it operates in Mode I or Mode II, the outage probability of the sensor in near cluster can be expressed as

(36) $$P_{out}^{near}=\left(1-P_{E_{2}}\right)P_{o}^{n}\left\{I\right\}+P_{E_{2}}\left(1-P_{r}\right)P_{o}^{n}\left\{III\right\}.$$

It is clear that $P_{o}^{n}\left\{\Psi_{I}\right\}=1$. The outage probability of Mode III is

(37) $$P_{o}^{n}\left\{III\right\}=P\left(\gamma_{D}^{n}<\theta_{d}\right)=F_{G_{I}}\left(\frac{r^{\beta }N_{0}\theta_{d}\left(1-\psi \right)}{B_{a}}\right),$$

where $F_{G_{I}}\left(\cdot \right)$ denotes CDF of exponential random variable $g_{I}$ with mean value $\bar{g_{I}}$ as $F_{G_{I}}\left(x\right)=1-exp\left(-\frac{x}{\bar{g_{I}}}\right)$. By substituting the terms derived above into Equation (36), we obtain the outage probability of the sensor in near cluster.

If the HAP transmit power $P_{0}$ is high enough, all sensors can be fully charged during the EH phase. Thus, if $P_{0}\to \infty$, $P_{E_{1}}=1$, $P_{E_{2}}=1$ and $P_{E_{3}}=1$ hold. By substituting these results into Equations (32) and (36), we can get outage probability of the sensor in the near and far cluster, respectively, if $P_{0}\to \infty$.

## 4. Simulation

In this section, we prove the correctness of the above analysis and expound how parameters have an effect on system performance. Specifically, sensors in the same cluster are so close to each other that we assign the same position for the sensor in the same cluster in proximity, and we assume that the HAP and the near cluster and the corresponding far cluster are formed into a line. We present all parameters in Table 1 and the value of important parameters have been labeled. Note that energy outage is the event that the sensor doesn’t have sufficient energy to perform information transmission.

**Figure 3:**
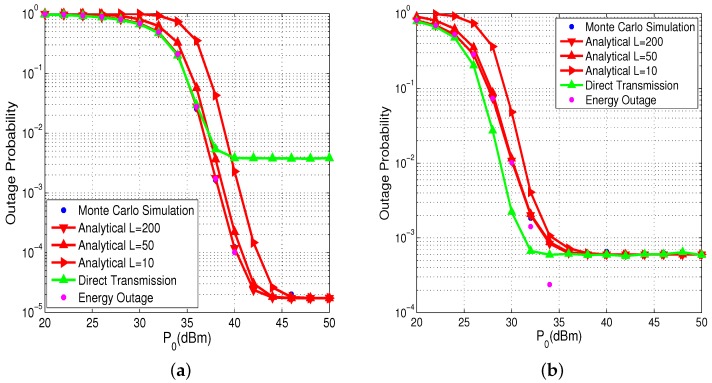
Outage probability of sensor in the far and near clusters versus the HAP transmit power $P_{0}$. (**a**) far cluster; (**b**) near cluster.

We first compare the derived theoretical outage probability of sensors in the far and near cluster with their corresponding Monte Carlo simulation results. Note that, in the Monte Carlo simulations, sensor battery level is continuous (i.e., L→∞). In Figure 3, we plot the theoretical outage probability curves of the proposed scheme with different battery levels. From these two figures, we can see that our analytic results coincide well with its corresponding Monte Carlo simulation results and curves getting closer as *L* increases. It is intuitive that, when L=200, two curves almost overlap, which confirms our theoretical analysis in Section 3. It means that L=200 can meet the requirement of the performance analyzing and there is no need to set a larger *L* that may incur huge computational complexity. When $P_{0}=50$ dBm, the outage probability curves of the sensor in far cluster with different *L* converge to the same outage floor, which is consistent to our derived theoretical results. Additionally, we can also get the conclusion that, when the HAP transmit power $P_{0}$ is relatively small, energy outage plays a dominant role in the outage performance. As $P_{0}$ becomes larger, there is almost no energy outage, and then information outage plays a dominant role. When $P_{0}$ is low, the sensor in near cluster has worse outage performance than direct transmission. This is because cooperative transmission consumes extra energy from the battery and may cause more energy outage. However, when $P_{0}$ is high enough, there is no difference between cooperative transmission and direct transmission in near cluster. On the contrary, the sensors in the cluster with the proposed scheme significantly outperform those with direct transmission schemes. That is to say, the proposed cluster cooperation scheme can improve the performance of the sensor in far cluster while the performance of sensors in near cluster is not sacrificed.

**Figure 4:**
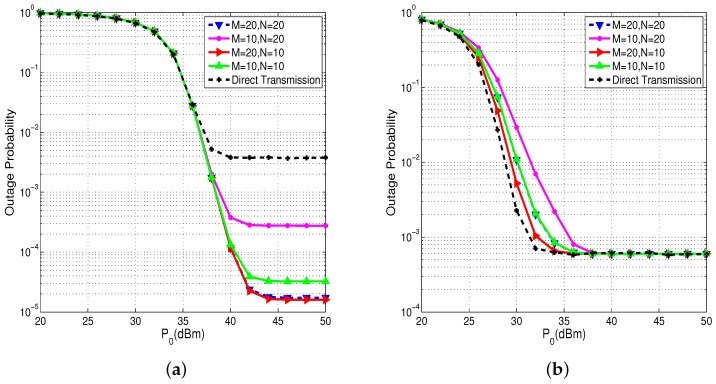
Outage probability of sensor in far and near cluster versus the HAP transmit power $P_{0}$ with different cluster scales. (**a**) far cluster; (**b**) near cluster.

The outage probabilities of sensors in far cluster and in near cluster with different sensor numbers are plotted in Figure 4. We can see that the numbers of sensors in the far and near cluster indeed affect the performance. From Figure 4a, we can see that when the HAP transmit power $P_{0}$ is quite high, the sensors in far cluster get lower and lower outage probability if the numbers of sensors in near cluster and in far cluster become larger. When M=20 and N=10, the sensor in far cluster gets the lowest outage probability and when M=10 and N=20, the sensor in far cluster gets the highest outage probability. The reason is that when *M* and *N* increase, the sensor in far cluster is more likely to request a sensor in near cluster for information relaying. We can also observe from Figure 4b that, when the HAP transmit power $P_{0}$ is quite low, the sensors in near cluster get smaller outage probability if the numbers of sensors in near cluster and in far cluster get larger. This is because, when *M* and *N* get larger, sensors in near cluster have more capabilities for relaying information for sensors in far cluster, which alleviates the probability of energy outage when $P_{0}$ is quite low. When $P_{0}$ becomes quite high, sensors in near cluster do not suffer from the lack of energy, so all curves tend to be the same outage floor. Moreover, we can still make the conclusion that cooperative transmission brings down the outage probability of sensors in the far cluster when the HAP transmit power $P_{0}$ exceeds 38 dBm without sacrificing the performance of sensors in near cluster.

**Figure 5:**
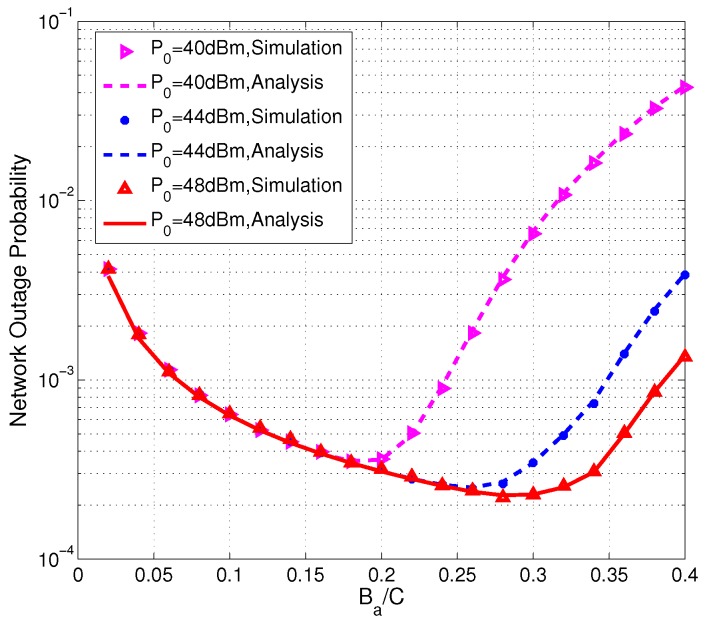
Network outage probability versus the normalized activation energy threshold $B_{a}/C$.

Figure 5 presents the simulation results and the theoretical values of the network outage probability versus the normalized activation energy threshold $B_{a}$ with different HAP transmit power $P_{0}=40,44,48$ dBm. It is obvious that there exists an optimal value $B_{a}$ that minimizes the network outage probability. In addition, the higher transmit power of the HAP, the larger the optimal value of $B_{a}$. The reason is that smaller $B_{a}$ results in a larger information outage probability, and, on the contrary, larger $B_{a}$ may cause too much energy consumption and lead to more energy outage. Moreover, $P_{0}$ increases, sensors can harvest more energy and could afford a larger energy threshold without causing more energy outage.

**Figure 6:**
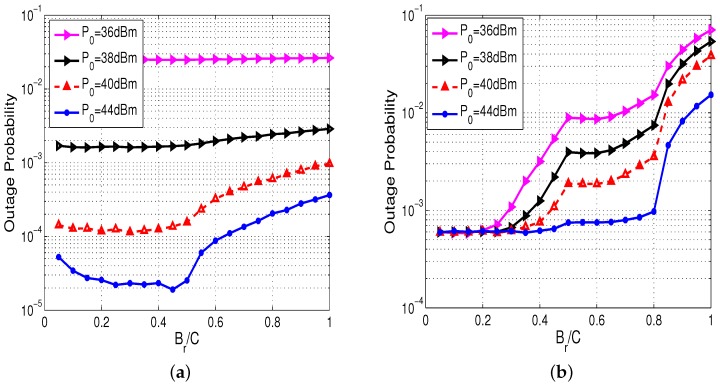
Outage probability of the sensor in the far and near cluster versus the relaying energy threshold $B_{r}$. (**a**) far cluster; (**b**) near cluster.

It is obvious that the outage probability of the sensor in far cluster would increase and that of the sensor in near cluster would decrease as $B_{r}$ increases. This is the reason that sensors in far cluster are more difficult to get assistance from the sensors in near cluster, and the sensors in near cluster are more likely to save their energy if we enhance relaying energy threshold $B_{r}$. Thus, we in this simulation let sensors in near cluster consume $B_{r}$ amount of energy for relaying information and show the results in Figure 6. We can observe from Figure 6a that, when $P_{0}=36$ dBm, the outage probability of sensors in far cluster almost remains constant. This is because energy outage plays a leading role in the outage performance, so that changing $B_{r}$ has nearly no effect on system performance. When $P_{0}$ is fairly high, we can see from Figure 6a that there exists an optimal threshold $B_{r}$ that minimizes the outage probability of sensors in far cluster. The reason is that smaller $B_{r}$ leads to less gain on cooperative transmission, and larger $B_{r}$ leads to more energy consumption of sensors in near cluster. We can also see that the higher $P_{0}$ is, the larger optimal value $B_{r}$ becomes. This is because higher $P_{0}$ means more energy can be harvested, and sensors in near cluster will consume more energy for information relaying. In Figure 6b, it is obvious that the outage probability of sensors in near cluster increases as $B_{r}$ grows. In addition, the lower $P_{0}$ is, the higher outage probability becomes. Comparing the two sub-figures, we can get the appropriate value of $B_{r}$ that can minimize the outage probability of sensors in far cluster and no longer sacrifice the performance of sensors in near cluster.

**Figure 7:**
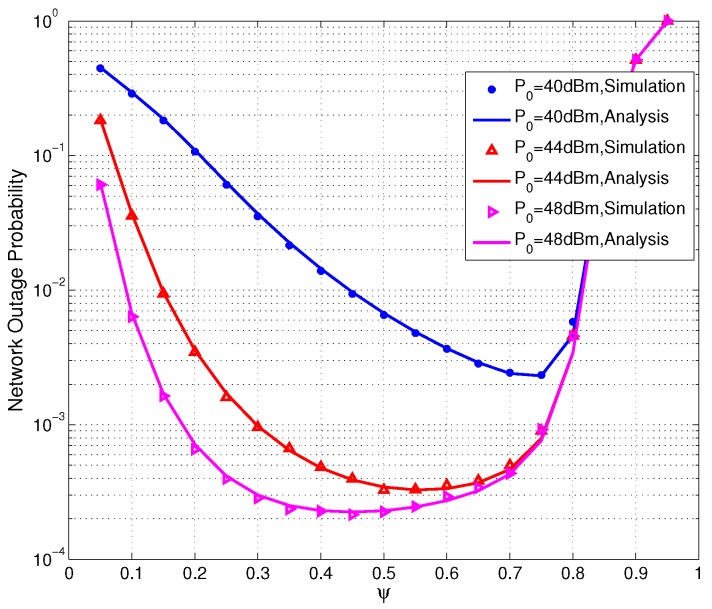
Network outage probability versus the time ratio ψ.

Figure 7 shows the network outage probability versus the energy harvest time ratio ψ with different HAP transmit power $P_{0}=40,44,48$ dBm. We can see from Figure 7 that there exists an optimal time ratio ψ that minimizes the network outage probability. When ψ is quite small, the harvested energy is too small in the sensors so that energy outage is very large. As ψ increases, energy outage becomes smaller and the network outage probability also becomes smaller. When ψ is large enough, energy outage barely happens, and then information outage plays the major role in the network outage event. If ψ still grows up , the spectral efficiency may be reduced, which causes worse performance in network outage probability. As $P_{0}$ increases, the optimal value of ψ decreases. This can be explained as two fold. First, as $P_{0}$ increases, sensors can harvest more energy. Secondly, the smaller ψ means higher spectral efficiency.

**Figure 8:**
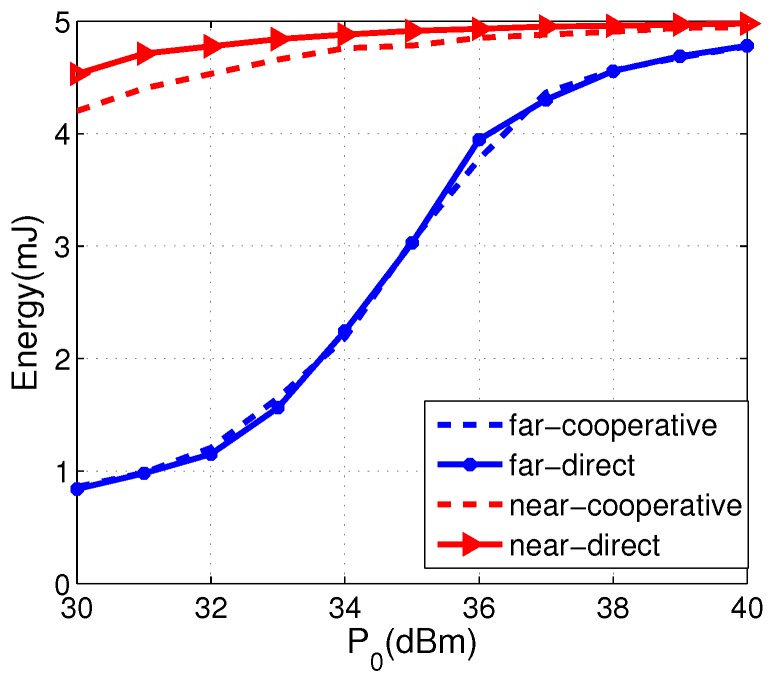
The average residual energy of sensors in the far cluster and near cluster versus the HAP transmit power $P_{0}$.

Due to the severe path loss, generally speaking, sensors in near cluster can harvest more energy than that in far cluster. However, in our system model, sensors in near cluster may consume extra energy for information relaying. In Figure 8, we plot the average residual energy of an arbitrary sensor in far cluster and near cluster, respectively. We can see that the residual energy of the sensor in far cluster in the proposed cooperative transmission is almost the same as that in direct transmission, and the residual energy of the sensor in near cluster in the proposed cooperative transmission is less than that in direct transmission. As $P_{0}$ increases, the differences between cooperative transmission and direct transmission becomes smaller. Thus, we can say that sensors in near cluster just use its abundant energy for information relaying, which brings about huge performance improvements on sensors in far cluster.

## 5. Conclusions

In this paper, we proposed that sensors in near cluster can act as relays and help the sensors in far cluster to forward information to the HAP in an amplify-and-forward (AF) manner if they don’t have their own information to transmit. By using a finite Markov chain to model the dynamic variation process of the sensor battery, we get the outage probability of sensors in far cluster and in near cluster, respectively. From simulation results, we can see that the outage probability of sensors in far cluster can be drastically reduced without sacrificing the performance of sensors in near cluster if the transmit power of HAP is fairly high. Furthermore, in the aspect of outage performance of far cluster, the proposed scheme significantly outperforms the direct transmission scheme without cooperation. Simulation results verify the correctness of theoretical analysis and show how parameters have an effect on system performance.

## Figures and Tables

**Table 1:** Theparamaeters of simulation.

Symbol	Definition	Value	Unit
R	Transmit Quantity of Data	1 [23]	
$P_{0}$	Transmit Power of HAP	20–50 [17]	dBm
α	Downlink Energy Path-Loss Exponent	2	
β	Uplink Information Path-Loss Exponent	2	
η	Coefficient of Energy Conversion	0.5 [23]	
ψ	Energy Harvest Time Ratio	0.5	
*N*	Number of Sensors in Far Cluster	20	
*M*	Number of Sensors in Near Cluster	20	
*C*	Battery Capacity of Sensor	5 [23]	mJ
*L*	Discrete Battery level	200	
$B_{1}$	Activation Energy Threshold	1 [23]	mJ
$B_{2}$	Relaying Energy Threshold	1 [23]	mJ
*r*	Distance between HAP and Near Cluster	20	m
*R*	Distance between HAP and Far Cluster	50	m
$d_{R}$	Distance between Far Cluster and Near Cluster	30	m
$\Omega_{1}$	Mean Value of Channel Gain $h_{e}$	1	
$\Omega_{2}$	Mean Value of Channel Gain $g_{e}$	1	
$\Omega_{3}$	Mean Value of Channel Gain $h_{I}$	1	
$\Omega_{4}$	Mean Value of Channel Gain $g_{I}$	1	
$\Omega_{5}$	Mean Value of Channel Gain $f_{I}$	1	
*p*	Probability of Sensor Transmitting Data	0.3	
$N_{0}$	Noise Power	−60 [23]	dBm

HAP: Hybrid Access Point.
